# Fluctuating Storage of the Active Phase in a Mn‐Na_2_WO_4_/SiO_2_ Catalyst for the Oxidative Coupling of Methane

**DOI:** 10.1002/anie.202004778

**Published:** 2020-06-17

**Authors:** Maximilian J. Werny, Yuanqing Wang, Frank Girgsdies, Robert Schlögl, Annette Trunschke

**Affiliations:** ^1^ Department of Inorganic Chemistry Fritz-Haber-Institut der Max-Planck-Gesellschaft Berlin Germany; ^2^ BasCat—UniCat BASF Joint Lab Technische Universität Berlin Germany

**Keywords:** catalysis, C−H activation, OCM, operando analysis, Raman spectroscopy

## Abstract

Structural dynamics of a Mn‐Na_2_WO_4_/SiO_2_ catalyst were detected directly under reaction conditions during the oxidative coupling of methane via in situ XRD and operando Raman spectroscopy. A new concept of fluctuating storage and release of an active phase in heterogeneous catalysis is proposed that involves the transient generation of active sodium oxide species via a reversible reaction of Na_2_WO_4_ with Mn_7_SiO_12_. The process is enabled by phase transitions and melting at the high reaction temperatures that are typically applied.

## Graphical abstract

**Give and take**: Operando Raman spectroscopy in combination with in situ XRD and thermal analysis was applied to study a Mn‐Na_2_WO_4_/SiO_2_ catalyst at work during the oxidative coupling of methane. Melting of Na_2_WO_4_ and Mn_7_SiO_12_‐MnWO_4_ redox chemistry occur simultaneously, thereby enabling the transient release and storage of active sodium oxide species. Coupling of these processes is essential to avoid catalyst deactivation.

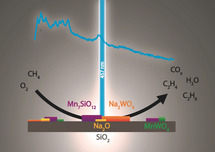

## Introduction

The oxidative coupling of methane (OCM) to ethane and ethene represents an attractive alternative to current crude‐oil‐based processes in order to access value‐added chemicals.^1^ Since the pioneering works of Keller and Bhasin,^2^ Hinsen and Baerns,^3^ and Ito and Lunsford^4^ in the early 1980s, the multi‐phase Mn‐Na_2_WO_4_/SiO_2_ catalyst has established itself as a high‐performance system that exhibits extended on‐stream stability at high reaction temperatures.^5^ Despite extensive investigations, both the active site and working mechanism of the catalyst remain much debated.^6^ In general, most research has been directed towards the structural characterization of the catalyst before and after the reaction or in a quenched state.^7^ Only limited efforts have, however, been made to elucidate the nature of the catalyst under working conditions.

In this report, a Mn‐Na_2_WO_4_/SiO_2_ catalyst (Supporting Information, Table S1, Figures S1–S3), synthesized in large scale by Simon et al.,5e was investigated with the aim of identifying structural motifs and phase transitions directly under relevant reaction conditions. A multi‐method approach, featuring electron microscopy (SEM‐EDX), thermal analysis (TG‐DTA‐MS), in situ and operando Raman spectroscopy and in situ X‐ray diffraction, was adopted. Reference compounds, in form of Na_2_WO_4_⋅2 H_2_O, MnWO_4_, and natural braunite (Mn_7_SiO_12_)^8^ were also examined (Supporting Information, Figures S4–S13). With pure Na_2_WO_4_ known to undergo a phase transition from solid to liquid at 695 °C (Supporting Information, Figure S11),^9^ the formation of a catalytically active liquid component, containing alkali and transition metal oxides, was investigated.

## Results and Discussion

**Figure 1:**
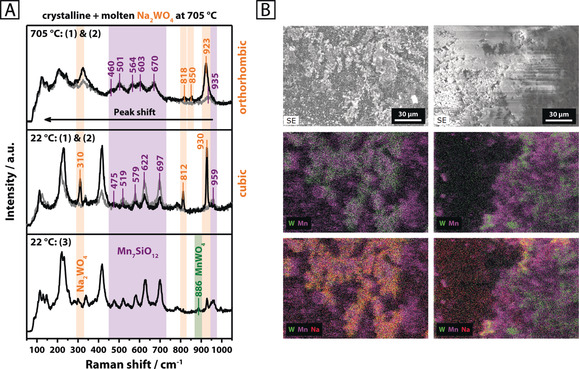
A) Raman spectra of Mn‐Na_2_WO_4_/SiO_2_ collected at three different spots using 457 nm excitation, as indicated in brackets, at room temperature (22 °C) and 705 °C in synthetic air (N_2_/O_2_=79/21); B) SEM‐EDX of two selected areas of the pristine Mn‐Na_2_WO_4_/SiO_2_ catalyst surface (Mn: purple, W: green, Na: red).

The as‐synthesized catalyst is a macro‐porous material with low specific surface area (2.9 m^2^ g^−1^) (Supporting Information, Figure S3). Its crystalline fraction is constituted by α‐cristobalite (92 wt %), quartz (0.5 wt %), Na_2_WO_4_ (4.1 wt %), and mixed‐valent Mn_7_SiO_12_ (3.4 wt %) (Supporting Information, Figure S1). The manganese silicate mostly contains the transition metal in the oxidation state +3 (Mn^2+^Mn^3+^_6_SiO_12_). Another phase, MnWO_4_, which only features Mn in the oxidation state +2, was sporadically detected at certain spots by Raman spectroscopy (Figure 1 A, bottom, Supporting Information, Figure S2). By investigating the aforementioned reference compounds using Raman spectroscopy (Supporting Information, Figures S4–S10), all observed bands were successfully allocated for the multi‐phase Mn‐Na_2_WO_4_/SiO_2_ catalyst system. The low concentration of MnWO_4_ is expected to arise from the higher stability of Mn_7_SiO_12_, relative to MnWO_4_, under oxidizing conditions. SEM‐EDX revealed an inhomogeneous distribution of manganese, sodium, and tungsten on the catalyst surface and inside the silica support, with Na_2_WO_4_ preferentially forming separate domains (appearing in yellow‐orange in Figure 1 B). Furthermore, large areas of the catalyst are only constituted by manganese, silicon and oxygen. Higher concentrations of Mn, in close vicinity to the Na_2_WO_4_ phase, may suggest a structural interaction of the Mn‐containing phases with Na_2_WO_4_. Freely existing W can also be observed, suggestive of silica‐supported WO_x_ species. However, typical bands for WO_*x*_ species dispersed on silica do not appear in the Raman spectrum of the catalyst (Figure 1 A).^10^ Surface inhomogeneities are also reflected in the Raman spectra recorded at different spots, which evidently feature varying concentrations of Na_2_WO_4_ and Mn_7_SiO_12_ (Figure 1 A, top and middle).

**Figure 2:**
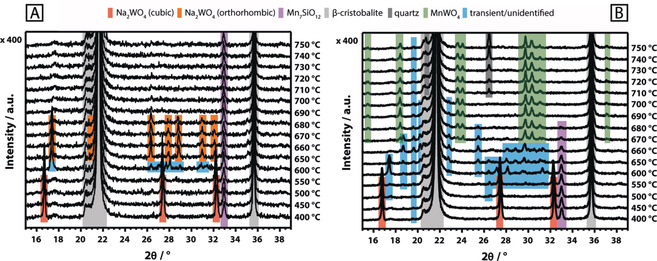
A) In situ XRD of the Mn‐Na_2_WO_4_/SiO_2_ catalyst recorded between 400 °C and 750 °C in He/O_2_ (He: 17.77 mln min^−1^, O_2_: 2.22 mln min^−1^); B) In situ XRD of the Mn‐Na_2_WO_4_/SiO_2_ catalyst recorded between 400 °C and 750 °C in He (20 mln min^−1^).

After testing the catalyst under relatively mild conditions (Supporting Information, Figure S14), a slightly increased concentration of quartz was observed, yet the general phase composition of the catalyst remained unaltered (Supporting Information, Figure S1). Both the support (in its high‐temperature β‐cristobalite modification ϑ>225 °C) as well as the Mn_7_SiO_12_ phase did not undergo structural changes in the temperature range of 400–750 °C in synthetic air (Figure 2 A; Supporting Information Figure S15). The Na_2_WO_4_ phase, on the other hand, displayed significant structural dynamics. When heating to 600 °C, the reflections of cubic Na_2_WO_4_ were replaced by an unidentified phase that is apparently formed during the transition of cubic to orthorhombic Na_2_WO_4_ (Figure 2 A; Supporting Information Figure S15). The latter was first observed at 650 °C and subsequently remained stable until 680 °C. Further heating to 690 °C resulted in the complete disappearance of the Na_2_WO_4_ reflections, which is suggestive of melting. This stands in agreement with the onset melting temperature of pure sodium tungstate (ϑ_m_=695 °C; Supporting Information, Figure S11). An endothermic event, indicated by a DTA signal at 689 °C in the thermal analysis of the catalyst in synthetic air (Supporting Information, Figure S16), further confirms the melting of Na_2_WO_4_ on the surface of the catalyst support. For the catalyst, the relatively small endothermic signal arises from the low concentration of Na_2_WO_4_ but is, nevertheless, clearly verified by its reversibility during cooling. Solidification of supported, molten Na_2_WO_4_ evidently results in the formation of an amorphous phase (Supporting Information, Figure S15). Furthermore, the crystallization of two unidentified phases is observed in the temperature range of 660–600 °C while cubic Na_2_WO_4_ only reappeared at 450 °C. Similar results were reported by Hou et al.,6j who observed a weakening of the reflections for Na_2_WO_4_ in air from 500 °C onwards and a complete disappearance at 700 °C. In addition to this, a significant broadening of the *ν_sym_* (W−O) stretching mode of Na_2_WO_4_ at 923 cm^−1^ was observed in the Raman spectrum of the catalyst above the melting point of Na_2_WO_4_ (Figure 1 A, top). Both, Takanabe et al.,6o and Yu. et al.,^11^ reported similar observations, describing a reversible disappearance and intensity loss of the characteristic Raman bands for Na_2_WO_4_ supported on TiO_2_ and CeO_2_, respectively. Raman spectroscopy at different sampling positions clearly proves that the melt does not completely wet the catalyst surface. While in some areas only signals of β‐cristobalite and Mn_7_SiO_12_ were evident (Figure 1 A, top, gray spectrum), other areas also featured a broadened spectrum of Na_2_WO_4_ (Figure 1 A, top, black spectrum). In summary, melting of the crystalline Na_2_WO_4_ phase in the Mn‐Na_2_WO_4_/SiO_2_ catalyst was clearly observed in synthetic air by in situ XRD and TG‐DTA analysis. However, according to Raman spectroscopy, a full wetting of the catalyst surface with molten Na_2_WO_4_ does not occur, that is, surface inhomogeneities clearly persist at temperatures higher than 700 °C.

**Figure 3:**
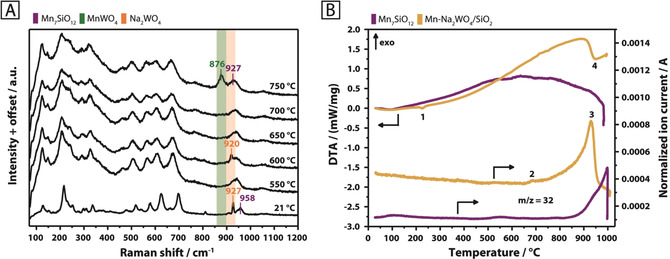
A) In situ Raman spectra of the Mn‐Na_2_WO_4_/SiO_2_ catalyst collected at room temperature (21 °C) and between 550 °C and 750 °C in N_2_ (20 mln min^−1^) using 457 nm excitation; B) TG‐DTA‐MS of the Mn‐Na_2_WO_4_/SiO_2_ catalyst (yellow) and Mn_7_SiO_12_ (purple) in Ar (70 and 100 mL min^−1^, respectively). The samples were subjected to a heating rate of 5 °C min^−1^ to reach a maximum temperature of 1000 °C. MS signal intensities were normalized to the carrier gas Ar (*m*/*z*=40).

In contrast to this, the structural evolution changes drastically in inert atmosphere. Cubic Na_2_WO_4_ disappears at 600 °C in favor of an unknown transient phase (Figure 2 B; Supporting Information, Figure S17), which only partially resembles the transient phase formed in air at 600 °C (Figure 2 A). Instead of detecting the formation of orthorhombic Na_2_WO_4_ at 650 °C, as observed in synthetic air (Figure 2 A), the patterns of the transient peaks are subject to further change. The intensity of the reflection near 33° 2*θ*, assigned to Mn_7_SiO_12_, starts to decrease, while peaks due to MnWO_4_ arise (Figure 2 B). The development of the MnWO_4_ peaks occurs simultaneously with the complete disappearance of the Mn_7_SiO_12_ reflection. A significant formation of MnWO_4_, by reaction of Mn_7_SiO_12_ with Na_2_WO_4_ and/or WO_*x*_, was also evident via Raman spectroscopy for nitrogen feed (Figure 3 A). The band of Mn_7_SiO_12_ at 958 cm^−1^ is no longer distinguishable at higher temperatures due to shift or broadening of the band of Na_2_WO_4_ at 927 cm^−1^. The latter is most likely caused by phase transition and melting of Na_2_WO_4_ or by formation of tetrahedrally coordinated, silica‐supported WO_*x*_ species. The disappearance of Mn_7_SiO_12_ and cubic Na_2_WO_4_ in the XRD, starting at 600 °C, is, therefore, most likely connected to a partial or complete conversion of the two phases to MnWO_4_. Thermal analysis of the Na_2_WO_4_, MnWO_4_, and Mn_7_SiO_12_ reference compounds (Supporting Information, Figures S11–S13) only revealed a significant oxygen release (*m*/*z*=32) at elevated temperatures for the Mn_7_SiO_12_ phase, with an onset at 807 °C (Figure 3 B; Supporting Information, Figure S13). Several different thermal events were observed for the Mn‐Na_2_WO_4_/SiO_2_ catalyst system in argon (Figure 3 B; Supporting Information, Figure S18). The DTA curve features an endothermic event (1) at 226 °C, which is assigned to the phase transition of the α‐cristobalite support to β‐cristobalite.^12^ Oxygen evolution from the catalyst is shifted to significantly lower temperatures when compared to the Mn_7_SiO_12_ reference, with the onset recorded at 653 °C (2). This could be associated with the phase transition of cubic Na_2_WO_4_ into unknown transient phases at 600 °C, the commencing formation of MnWO_4_ at 650 °C as well as the disappearance of Mn_7_SiO_12_, also observed from 650 °C onwards (Figure 2 B). Instead of a well‐defined endothermic peak, the DTA curve only displays minor irregularities in the temperature regime between 600 °C and 700 °C (Figure 3 B; Supporting Information, Figure S18). It is possible that the superposition of the melting and redox processes, as observed via in situ XRD in the absence of air in this temperature range, limits the ability of DTA to clearly detect melting.

Progressive surface mobility, initiated by restructuring of supported Na_2_WO_4_ above 600 °C and facilitated by melting of residual Na_2_WO_4_ at higher temperatures, can enable the reaction between Mn_7_SiO_12_ and Na_2_WO_4_ as shown in Equation (1). This, in turn, leads to the reduction of Mn^3+^ (in Mn_7_SiO_12_) to Mn^2+^ (in MnWO_4_), the release of molecular oxygen with its maximum at 927 °C ((3) and (4) in Figure 3 B), and the formation of amorphous or dispersed sodium oxide.

(1) $$\mathrm{Mn}{}^{2+}\mathrm{Mn}{}^{3+}{}_{6}\mathrm{SiO}{}_{12}\;+\;7\;\mathrm{Na}{}_{2}\mathrm{WO}{}_{4}\;↔7\;\mathrm{MnWO}{}_{4}\;+\;7\;\mathrm{Na}{}_{2}O\;+\;\mathrm{SiO}{}_{2}\;+\;1.5\;O{}_{2}$$

The broad temperature range for the oxygen release covers the onset temperature of oxidative coupling of methane (650 °C) as well as the reaction temperatures that are typically applied (700–800 °C). A weight loss of 0.2 % (0.22 mg) was caused by the event, with the DTA curve indicating endothermicity (4). It is postulated that the process described in Equation (1) provides mobile lattice oxygen under steady‐state operation of the catalyst. This is in agreement with O_2_‐TPD experiments performed by Gordienko et al.,^13^ who identified two forms of lattice oxygen that may potentially contribute to catalysis upon desorption at relevant temperatures. The source of oxygen has been attributed to manganese oxide,6j, 7 or any unspecified lattice oxygen.^14^ Potentially more decisive is the release of an active form of sodium oxide species that has been proposed to catalyse the oxidative coupling of methane by generation of OH radicals at high temperatures,6l, 6m, 6o presumably under involvement of homogeneous gas‐phase reactions^15^ due to the high volatility of sodium compounds under operation conditions.^16^

**Figure 4:**
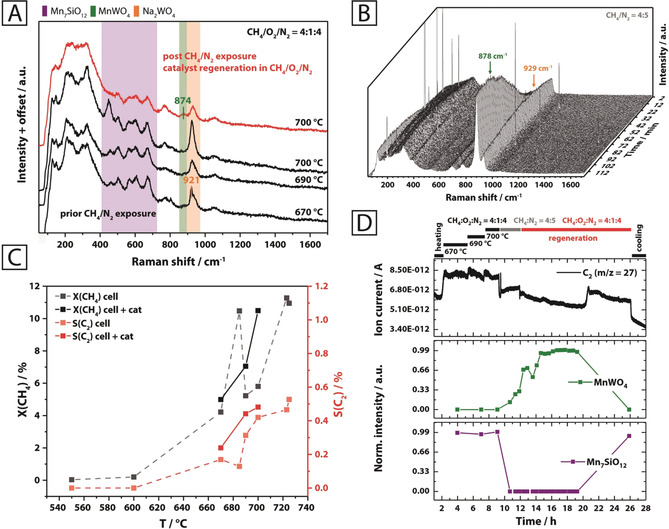
Operando Raman experiment conducted on the Mn‐Na_2_WO_4_/SiO_2_ catalyst using 457 nm excitation. The catalyst was studied in a temperature range of 670–700 °C under CH_4_/O_2_/N_2_=4:1:4 (A, black spectra), followed by exposure to CH_4_/N_2_=4:5 at 700 °C (B) and subsequent regeneration under CH_4_/O_2_/N_2_=4:1:4 at 700 °C (A, red spectrum) (total flow=10 mL min^−1^; W/F=0.0030 g min mL^−1^). The Raman spectra under steady‐state conditions (A) were collected using an exposure time of 30 min. X(CH_4_) and S(C_2_) indicate that the catalyst was active in the given temperature regime (C). By comparing the collected mass spectrometry data that is representative for C_2_ formation and the band intensities for Mn_7_SiO_12_ (670 cm^−1^) and MnWO_4_ (878 cm^−1^), the re‐formation of Mn_7_SiO_12_ from MnWO_4_ was found to be associated with an increase in catalytic activity during the catalyst regeneration phase.

Evidence, that the redox process described in Equation (1) occurs under reaction conditions and is indeed reversible, was provided by operando Raman spectroscopy at different reaction temperatures and feed compositions (Figure 4; Supporting Information Figure S19). In all operando spectra (Figures 4 A and B), the region of 100–400 cm^−1^ is dominated by a broad band of the β‐cristobalite support, which is formed via phase transition from α‐cristobalite at 225 °C.12a, 12c, 17 The spectra recorded under steady‐state conditions in a CH_4_/O_2_/N_2_ (4:1:4) feed at various temperatures (Figure 4 A) predominantly feature bands of β‐cristobalite and the remaining steady‐state concentrations of Mn_7_SiO_12_ (450–700 cm^−1^) and Na_2_WO_4_ (920–930 cm^−1^). At the highest reaction temperature, the formation of MnWO_4_ becomes apparent, as indicated by a very weak band at 874 cm^−1^. Carbon oxides, ethane, and water were identified as the main reaction products via online mass spectrometry and gas chromatography in the effluent gas of the Raman cell (Supporting Information, Figures S20,S21). Significant formation of coke was not observed (Figure 4 A). The selectivity to C_2+_ products was much lower compared to analogous experiments conducted in a quartz fixed bed reactor without dilution of the catalyst (Supporting Information, Figure S14), which is attributed to consecutive reactions of the desired C_2+_ products on the hot stainless‐steel walls of the operando cell and the inadequate reactor geometry. However, comparison of the catalytic tests conducted with the empty operando cell and the same cell filled with the catalyst under identical reaction conditions clearly showed a lower activity and selectivity to C_2+_ products for the empty reactor (Figure 4 C).

Changes to the spectral composition were observed upon switching to a reducing CH_4_/N_2_ (4:5) feed at 700 °C (Figure 4 B). This was accompanied by a significant decline in catalyst performance and the formation of hydrogen and carbon monoxide as main products, suggesting that lattice oxygen is consumed and gas‐phase reactions as well as methane pyrolysis prevail under these conditions (Supporting Information, Figures S20 and Figure S21). The complete removal of oxygen leads to the formation of MnWO_4_, as indicated by the band at 878 cm^−1^ (Figures 4 B and D). Thus, a reaction of Na_2_WO_4_ and/or WO_x_ with Mn_7_SiO_12_ and/or MnO_*x*_ to yield MnWO_4_ occurs under reducing conditions. The formation of coke is excluded here based on Raman spectroscopy (Figure 4 A). During catalyst regeneration using the initial reaction feed (CH_4_/O_2_/N_2_=4:1:4), braunite is suddenly reformed at the expense of MnWO_4_ after approximately 8 h (Figure 4 D). Moreover, due to this reversible phase transformation, the formation of C_2_ products is re‐initiated (Supporting Information, Figure S20 and Figure S21 after 8 h), thereby highlighting the significance of the Mn_7_SiO_12_ phase in maintaining catalytic activity. The presence of Mn_7_SiO_12_ is also clearly responsible for the formation of CO_2_ in place of CO (Supporting Information, Figure S21). As can be seen in Figure 4 A, the spectrum initially observed under steady‐state conditions, is restored entirely by switching back to a CH_4_/O_2_/N_2_ feed (Figure 4 A, red spectrum).

## Conclusion

**Scheme 1:**
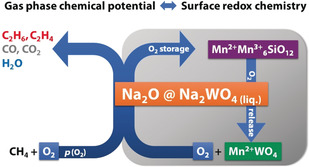
Impeded phase transition: Transient release of the active phase controlled by the partial pressure of oxygen in the gas phase and the surface redox chemistry.

In conclusion, the present in situ and operando experiments disclosed reversible redox activity of the Mn_7_SiO_12_, MnWO_4,_ and Na_2_WO_4_ phases under operation conditions in the oxidative coupling of methane over Mn‐Na_2_WO_4_/SiO_2_. A new concept is proposed that involves the fluctuating storage and release of an active phase in heterogeneous catalysis. According to Equation (1), active sodium oxide species, which are responsible for high activity and selectivity in the oxidative coupling of methane,6o are generated in the catalytically relevant temperature regime in small amounts. The extent of this reaction is controlled by the oxygen partial pressure in the gas phase and the redox chemistry on the surface (Scheme 1). While the structural synergy of all phases is responsible for the high stability and activity of the catalyst, the supported Na_2_WO_4_ phase acts as storage phase responsible for transient generation of active sodium oxide species that would, in absence of the stabilizing Mn_7_SiO_12_–MnWO_4_ redox couple, suffer from steady sublimation,^16^ thus leading to catalyst deactivation.6b, 6g As long as the oxygen partial pressure in the reactor is not too low, a small steady‐state concentration of the active phase is formed according to Scheme 1.

Phase transitions and melting of Na_2_WO_4_ enable the generation of the active phase by providing mobile sodium species. Furthermore, the supported Mn_7_SiO_12_ phase was observed to function as oxygen‐donor at working temperatures, which further enhances sodium oxide formation and, thus, has implications for the reactivity. However, the availability of adsorbed or lattice oxygen due to the presence of redox‐active elements alone6j, 7, 13, 14 cannot be responsible for the outstanding performance of the Mn‐Na_2_WO_4_/SiO_2_ catalyst and does not explain the mechanistic role and importance of Na in this system. On the other hand, a pure silica‐supported sodium oxide would rapidly deactivate under the severe reaction conditions applied in the oxidative coupling of methane.6g Only the chemical complexity of the Mn‐Na_2_WO_4_/SiO_2_ catalyst guarantees long‐term stability. The synergistic element combination discovered by chance is so successful because melting and redox reactions occur in the same temperature window (Figure 2 and Figure 3).

In the presence of gas‐phase oxygen, the phase transition of Mn_7_SiO_12_ and Na_2_WO_4_ into Na_2_O and MnWO_4_ [Eq. (1)] is largely impeded and generates only transient amounts of active6o sodium oxide species. Apparently, only a small concentration of oxygen is necessary to keep the system in this highly active state (>88 % oxygen conversion in the steady state, see Supporting Information, Figure S21, *t*>8 h). Such a low concentration of oxygen in the gas phase is beneficial in terms of the selectivity. Only in total absence of oxygen is the system disturbed and MnWO_4_ formed in noticeable amounts. The oxygen donor Mn_7_SiO_12_ (Figure 3 B), however, evidently hinders a complete and rapid transformation into MnWO_4_ and Na_2_O (Figure 4 B), which would lead to a loss of Na_2_O due to sublimation and progressive catalyst deactivation. The described scenario may also be considered as displacement of the redox chemistry from the organic to the inorganic part of the hybrid reaction system. Therefore, in future concepts of catalyst design it might be reasonable to take into account that the activation of methane could also proceed via an acid‐base reaction by using the strong base O^2−^ as catalyst avoiding radical chemistry in the selective pathway.

Our experiments clearly show that MnWO_4_ is a product of catalyst deactivation, which is formed under strongly reducing reaction conditions. However, regeneration by increasing the partial pressure of oxygen in the feed again is possible (Figures 4 D and Supporting Information, Figure S21).

The study in this report is an example for how in situ and operando Raman spectroscopy techniques can be applied as effective, non‐invasive tools to obtain valuable information on high‐temperature catalysts under relevant operation conditions. Based on experimental evidence, it is clearly explained how the chemical complexity of the Mn‐Na_2_WO_4_/SiO_2_ catalyst warrants a high yield of C_2_ products and long‐term stability in the oxidative coupling of methane.

## Conflict of interest

The authors declare no conflict of interest.

## Supporting information

As a service to our authors and readers, this journal provides supporting information supplied by the authors. Such materials are peer reviewed and may be re‐organized for online delivery, but are not copy‐edited or typeset. Technical support issues arising from supporting information (other than missing files) should be addressed to the authors.

Supplementary
