# Supplementary material for: Fluctuating Storage of the Active Phase in a Mn‐Na2WO4/SiO2 Catalyst for the Oxidative Coupling of Methane
Source: Angew Chem Int Ed Engl. 2020 Jun 17;59(35):14921–6. doi: 10.1002/anie.202004778 (PMC7496389; doi:10.1002/anie.202004778)
Supplement: Supplementary file 1 — Supplementary [file ANIE-59-14921-s001.pdf]

Angewandte Chemie
Eine Zeitschrift der Gesellschaft Deutscher Chemiker

Supporting Information

Fluctuating Storage of the Active Phase in a Mn-Na_2WO_4/SiO_2 Catalyst for the Oxidative Coupling of Methane

*Maximilian J. Werny, Yuanqing Wang, Frank Girgsdies, Robert Schlögl, and Annette Trunschke**

anie_202004778_sm_miscellaneous_information.pdf

CONTENT

1 CATALYST PREPARATION 1

2 REFERENCE SAMPLES 1

3 CATALYST CHARACTERIZATION 2

4 ACTIVITY TESTING 4

5 SUPPLEMENTARY FIGURES AND TABLES 5

6 REFERENCES 16

1 Catalyst preparation

The Mn-Na_2WO_4/SiO_2 catalyst was prepared and supplied by Simon *et al*. (Technische Universität Berlin). The catalyst was synthesized following a nominal composition of 2.0 wt% Mn^{2+} ions and 4.5 wt% Na_2WO_4 on SiO_2 and processed as concentring granulate using a fluidized bed granulator (Büchi 710, Switzerland). Amorphous SiO_2 with grain sizes ranging from 250−350 µm (Sigma-Aldrich, Silica Gel, Davisil, Grade 634), was used as catalytic support material. The fluidizing chamber of the granulator was loaded with 200 g of these nuclei. An air flux of 30 m^3/h, heated up to 80 °C, was used. As a result, the granules underwent fluidization. The atomisation column for coating consisted of a vertically assembled two-component nozzle (inner tube for guiding solutions and outer coaxial gas outlet). The nozzle was operated at 80 °C at a pressure of 0.07 MPa (0.7 bar). Aqueous solutions of $Mn(NO_3)_2{\cdot}4H_2O$ and $Na_2WO_4{\cdot}2H_2O$, henceforth referred to as Mn-solution and Na-W-solution, were prepared at 70 °C in the desired concentrations. After the start up process, the aqueous Mn-solution was sprayed into the fluidizing chamber, resulting in a primary coating of Mn phases on the SiO_2 granules. Using the Na−W-solution, a second layer was subsequently applied. The concentrically coated SiO_2-granules were then heated up to 250 °C in an Al_2O_3-cruicible under air for 1 h, resulting in the removal of residual water and remaining nitrates. Finally, the coated SiO_2 precursors were annealed at 800 °C for 8 h under air. All annealing procedures were performed using a heating rate of 10 K/min. No additional activation procedures were performed before analysis.

2 Reference samples

$Na_2WO_4{\cdot}2H_2O$ and $MnWO_4$ were used as received from commercial sources (Sigma-Aldrich, 99%, LOT: 11725JD and Alfa Aesar, 99.9%, LOT: Y25AO43) without undergoing any further purification. The natural Mn_7SiO_{12} mineral (origin: Langban, Sweden) was supplied by Dr. Rupert Hochleitner (Mineralogische Staatssammlung München). The mineral was ground using a planetary mill. No additional purification procedures were applied.

3 Catalyst characterization

Scanning electron microscopy (SEM) was performed with a Hitachi S-4800 field emission scanning electron microscope operating in the range of 0.1 to 30 kV (acceleration voltage) in secondary electron (SE) mode. The instrument is equipped with a cold field emission gun and produces a maximum resolution of 1.0 nm (STEM mode). Morphology studies were performed at a voltage of 1.5 kV and a working distance of 3.0 mm. For this, the sample was applied to double-sided adhesive, conductive carbon tape, which was then stuck onto a carbon disc to reduce the background signal. The energy dispersive X-ray analysis data was collected with an integrated Bruker EDX QUANTAX 800 system (with XFlash®6) featuring a SDD (silicon drift detector) detector. For X-ray analysis, an excitation voltage of at least two times the highest peak energy was applied for the elements of interest. The voltage was set at 20 kV for all EDX measurements, resulting in an average interaction depth of 2.8 µm. The working distance for the detection of X-rays was optimized to equal 10.0 mm due to the geometry of the system.

Thermogravimetric studies were conducted using a NETZSCH STA 449 C *Jupiter* apparatus. The system was connected to a Pfeiffer Vacuum ThermoStar GSD 301 T for additional mass spectrometry analysis. 104.5 mg of the Mn-Na_2WO_4/SiO_2 catalyst were placed in a DTA sample holder and pre-treated in synthetic air (21% O_2 in Ar, 100 mL/min) at 500 °C for 1 h (heating rate: 10 °C/min). H_2O, CO_2 and carbonaceous deposits were consequently removed from the surface. For the actual TG-DTA measurement, the sample was heated to 1000 °C at a continuous heating rate of 5 °C/min in Ar (total flow rate = 70 mL/min). The temperature was maintained for 1 h. The reference samples were all analysed according to a similar procedure. 70.4 mg of $Na_2WO_4 \cdot 2H_2O$ were placed in a DSC sample holder and heated to 780 °C at a heating rate of 10 °C/min in synthetic air (21% O_2 in Ar, 100 mL/min). In the case of Mn_7SiO_{12}, 17.6 mg of the sample were heated to 1000 °C in a DSC sample holder at a heating rate of 5 °C/min in Ar (100 mL/min). The temperature was maintained for 1 h. For the characterisation of $MnWO_4$, 31.2 mg were placed in a DSC sample holder and heated to 1000 °C at a heating rate of 5 °C/min in Ar (100 mL/min). The maximum temperature was maintained for 1 h.

Raman spectroscopy was performed using a customized micro Raman spectroscopic system assembled by S&I Spectroscopy & Imaging GmbH (Warstein, Germany). The experimental setup consists of an optical microscope (Olympus) and a triple-spectrometer (TriVista TR 557, Princeton Instruments) combined with a liquid nitrogen cooled CCD detector (PyLoN:2K, Princeton Instruments). A single-stage configuration was adopted for all measurements. As excitation source, a 457 nm single frequency continuous-wave diode-pumped laser from Cobolt was used for all measurements. Two neutral density filters were installed to tune the laser power and an edge filter to cut the laser line from the scattered light. Unless stated otherwise, a laser power of ~0.5 mW, a 600 grooves/mm grating and a 100 µm slit entrance were applied. A ×10 microscope objective was used to focus the incident beam on the sample. For spectrometer frequency calibration, a silicon wafer (520.7 ± 0.5 cm^{-1}) was applied. The sample position was maintained if not stated otherwise. The *in situ* measurements in O_2/He and O_2/N_2 feed were conducted in a commercial CCR1000 reaction cell from Linkam Scientific

Instruments, using 10 mg of sample. *Operando* measurements in $CH_4/O_2/N_2$ were performed in a HVC-MRA-5 cell from Harrick Scientific Instruments using approximately 30 mg of sample. The outgoing gasline of the Harrick cell was connected to a micro-GC (Varian CP-4900 or Agilent 490 Micro GC) and a quadrupole mass spectrometer (Pfeiffer) performing on-line gas product analysis.

Elemental analysis via **wavelength dispersive X-ray fluorescence spectrometry (XRF)** was performed using a Bruker S4 Pioneer spectrometer. It is equipped with a Rh end window X-ray tube (20−60 kV, 5−150 mA, max. 4 kW) and proportional and scintillation counters for the detection of lighter and heavier elements, respectively. 8.90 g di-lithium tetraborate (> 99.995%, Aldrich) were added to 97.2 mg of the Mn-Na_2WO_4/SiO_2 catalyst for fluxing digestion in a gold hardened Pt crucible at 1300 °C (Vulcan Fusion Machine, HD Elektronik & Elektrotechnik GmbH). A pellet with a 40.0 mm diameter was obtained for analysis.

Elemental analysis via **inductively coupled plasma optical emission spectroscopy (ICP-OES)** was performed using an Optima 8300 ICP-OES system by Perkin Elmer. 10.0 mg LiF were added to 10.0 mg of the catalyst. The mixture was then dissolved in 10 mL HNO_3 (65%, Suprapur) and heated to 220 °C (60 bar, 2 h) in a microwave autoclave. The resulting solution was filled up to 50 mL with ultra-pure water and diluted again by a factor of ten for analysis.

Nitrogen adsorption/desorption was studied at −196 °C using a Quantachrome Autosorb-6B-MP system after outgassing the Mn-Na_2WO_4/SiO_2 catalyst (53.7 mg) in vacuum for 12 h at 150 °C. All data treatments were performed using the Quantachrome Autosorb software package. The specific surface area S_{BET} was calculated according to the multipoint BET method in the p/p_0=0.06–0.29 pressure range, assuming a N_2 cross sectional area of 16.2 Å^2. An adsorption/desorption isotherm consisting of 79 measurement points was recorded in the p/p_0 range of 0.04−1.0 to evaluate the microporosity of the sample.

All **X-ray diffraction (XRD)** measurements were performed in Bragg-Brentano geometry on a Bruker AXS D8 Advance II theta/theta diffractometer, using Ni-filtered Cu Kα radiation and a position sensitive energy-dispersive LynxEye silicon strip detector. The mortared samples were filled into the recess of a cup-shaped sample holder, with the surface of the powder bed being in alignment with the sample holder edge (front loading). The measurement range was 6.0° to 140.0° (2θ). Qualitative phase identification was performed using the DIFFRAC.EVA software (Bruker AXS, 2010−2016) by matching the powder XRD patterns against the PDF-4+ database (ICDD, 2017). Phase quantification is based on whole pattern fitting according to the Rietveld method, using the TOPAS 5.0 program (Bruker AXS, 2014). The *in situ* XRD data was collected on a STOE Theta/theta X-ray diffractometer (Cu Kα_{1+2} radiation, secondary graphite monochromator, scintillation counter) equipped with an Anton Paar XRK 900 *in situ* reactor chamber. The measurement range was 15.0−39.0° (2θ). A step size of 0.02° and a counting time of 15 s were used. The gas feed was mixed by means of Bronkhorst mass flow controllers, using helium as inert balance gas at a total flow rate of 20 mLn/min. The effluent gas composition was monitored with a Pfeiffer OmniStar quadrupole mass spectrometer. The *in situ* experiment under oxidizing conditions was conducted in a feed of O_2:He = 1:8 (total flow rate = 20 mLn/min). 64.0 mg of mortared catalyst were used. The sample was directly heated from 25 °C to

400 °C at a heating rate of 20 °C/min. From thereon, a heating rate of 10 °C/min was applied. Temperature steps of 50 °C were maintained until 650 °C. The range of 650−750 °C was covered in 10 °C steps. An analogous temperature program was maintained during cooling. The experiment under inert conditions was performed in pure He (total flow = 20 mLn/min) following an identical temperature program. 60.0 mg of mortared catalyst were used.

4 Activity testing

The bulk catalytic activity of the Mn-Na_2WO_4/SiO_2 catalyst in the oxidative coupling of methane was studied by using a setup for partial oxidation (Integrated Lab Solutions) with eight fixed-bed quartz reactors (6 mm inner diameter) in parallel. Each reactor was equipped with a thermocouple for measuring the temperature inside the catalyst bed. The catalytic performance was determined at atmospheric pressure under steady state conditions. The applied temperature range for the reaction was 700−750 °C. A maximum reaction temperature of 750 °C was maintained to avoid catalyst decomposition. The reactor was first heated up to 700 °C under reactant gas flow, followed by a stepwise increase to 725 °C and 750 °C respectively (heating rate = 5 °C/min). The reactant feed comprised CH_4, O_2 and He as diluent in a ratio of 4:1:4 (total flow rate = 40 mL/min). The Mn-Na_2WO_4/SiO_2 catalyst was sieved to a particle size of 250−355 mm. 100 mg of the catalyst were loaded into the reactor without prior dilution for direct comparison with the *operando* Raman experiments that were performed thereafter. A contact time variation was performed at a fixed temperature of 750 °C by variation of the gas flow (40/60/80 mL/min). The calculated pressure drop was below 0.5 mbar for all loadings. An online gas chromatograph (Agilent 7890A) was used for gas analysis. A combination of Plot-Q (length 30 m, 0.53 mm internal diameter, 40 mm film thickness) and Plot-MoleSieve 5A columns (30 m length, 0.53 mm internal diameter, 50 mm film thickness), connected to a thermal conductivity detector (TCD), was used to analyse the permanent gases CH_4, O_2, CO, CO_2 and He. A system of a FFAP (length 30 m, 0.53 mm internal diameter, 1 mm film thickness) and a Plot-Q column (length 30 m, 0.53 mm internal diameter, 40 mm film thickness), connected to a flame ionization detector (FID), was used to analyse C_2−C_3 hydrocarbons and oxygenates. The carbon balance was always at 100%. For all experiments, the conversion of methane X and the product selectivity S_k were calculated based on the sum of products as follows [Eq. (1) and (2)]:

$$X = \frac{\sum_{i=1}^{n} \frac{n_i(\text{product})}{|v_i|}}{\sum_{j=1}^{k} \frac{n_j(\text{C-compound})}{|v_j|}} \tag{1}$$

$$S_k = \frac{N_k(\text{C-atoms}) \times n_k(\text{product})}{\sum_{i=1}^{n} N_i(\text{C-atoms}) \times n_i(\text{product})}, k = 1 \dots n \tag{2}$$

The formulas were also applied in the calculations for the *operando* Raman experiments.

5 Supplementary Figures and Tables

Table S1: Catalyst composition as determined via ICP-OES (*) and XRF analysis (**).

Element	Nominal Composition [wt%]	Concentration [wt%]
Mn	2.00	1.94 *
Na	0.70	0.65 *
W	2.82	2.70 ± 0.02 **
Si	44.17	42.79 ± 0.16 **

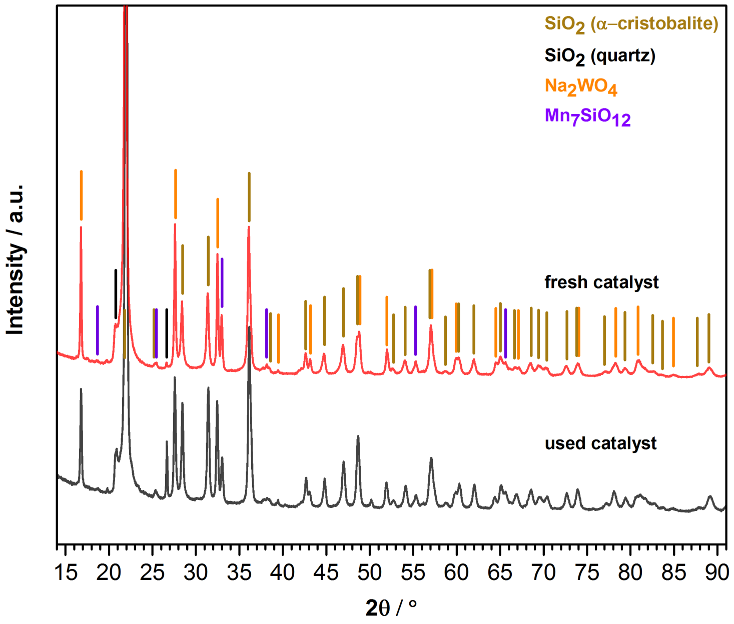

Figure S1. X-ray diffraction pattern of the Mn-Na_2WO_4/SiO_2 catalyst at room temperature. Quantitative phase analysis revealed the fresh catalyst to be constituted by 92.01 wt% α-cristobalite, 0.49 wt% quartz, 4.11 wt% Na_2WO_4 and 3.38 wt% Mn_7SiO_{12} (based on observed crystalline phases). Quantitative phase analysis of the catalyst after catalytic testing revealed a phase composition of 89.82 wt% α-cristobalite, 3.21 wt% quartz, 3.88 wt% Na_2WO_4 and 3.09 wt% Mn_7SiO_{12} (based on observed crystalline phases).

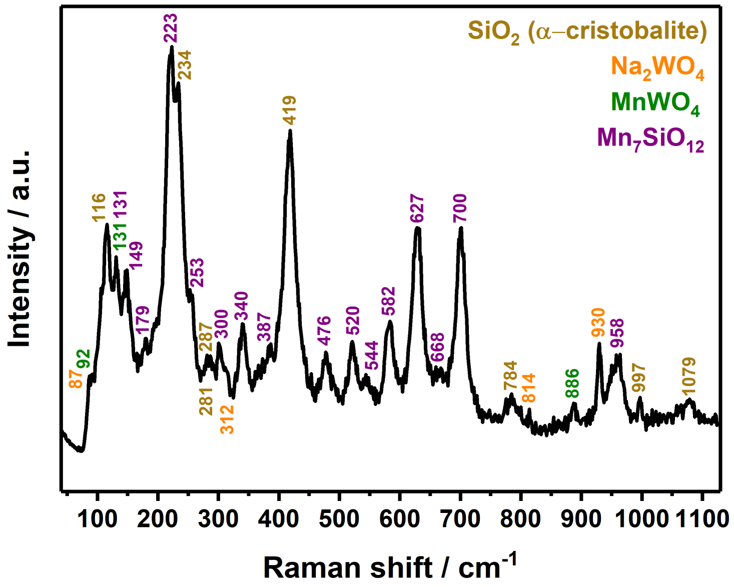

Figure S2. *In situ* Raman spectrum of the Mn-Na_2WO_4/SiO_2 catalyst recorded at 457 nm (He:O_2 = 79:21, total flow = 10 mL/min, T = 22 °C, exposure time = 10 min); assignment of peaks originating from α-cristobalite based on literature.[2]

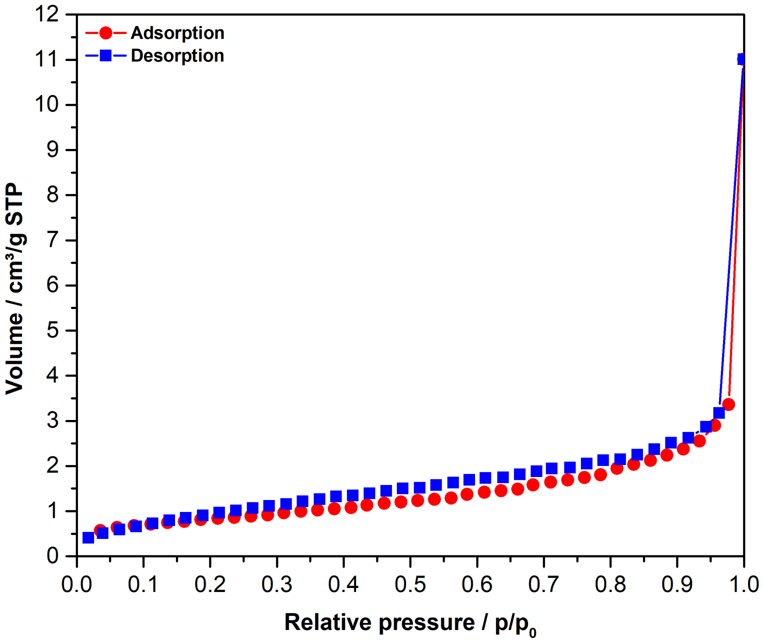

Figure S3. N_2-adsorption/desorption isotherm for the Mn-Na_2WO_4/SiO_2 catalyst (53.7 mg). A surface area of S_{BET} = 2.9 m²/g was determined.

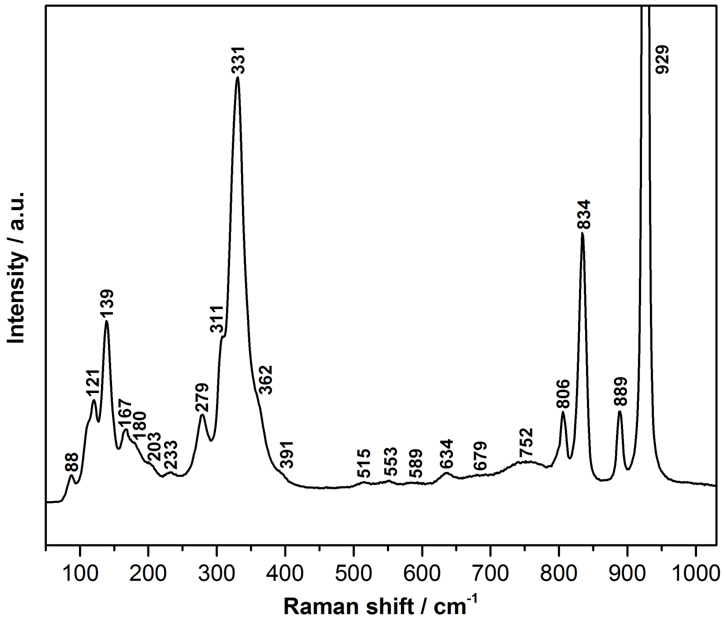

Figure S4. *Ex-situ* Raman spectrum of pure $Na_2WO_4 \cdot 2H_2O$ recorded in static air at 23 °C using a 457 nm laser (exposure time = 1.5 min).

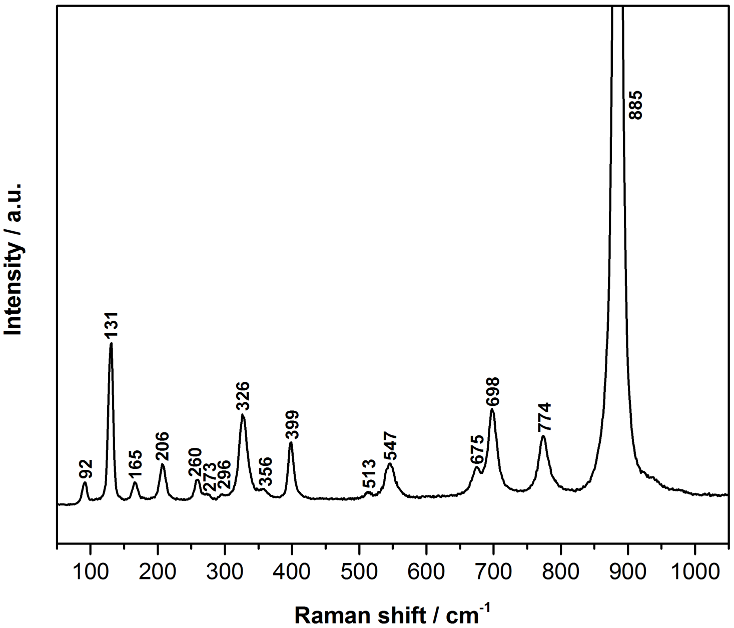

Figure S5. *Ex-situ* Raman spectrum of pure $MnWO_4$ recorded in static air at 23 °C using a 457 nm laser (exposure time = 3 min).

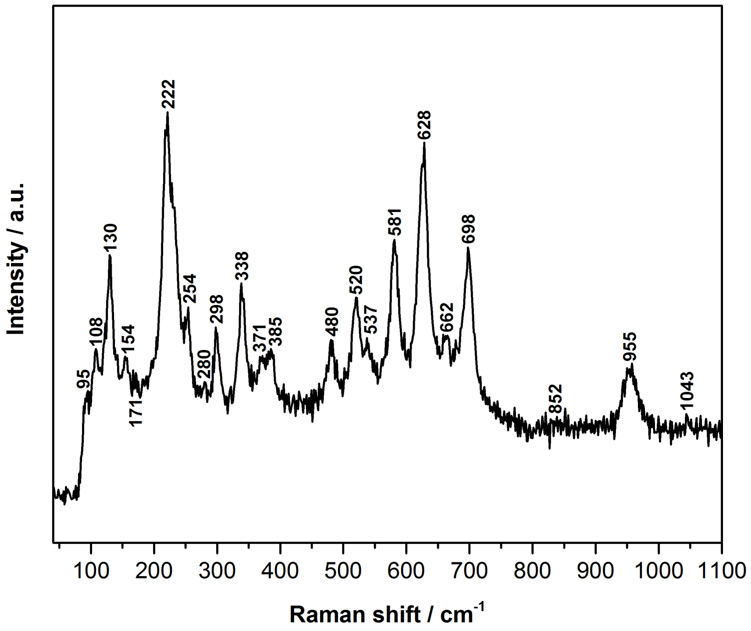

Figure S6. *Ex-situ* Raman spectrum of pure Mn_7SiO_{12} recorded in static air 23 °C using a 457 nm laser (exposure time = 10 min).

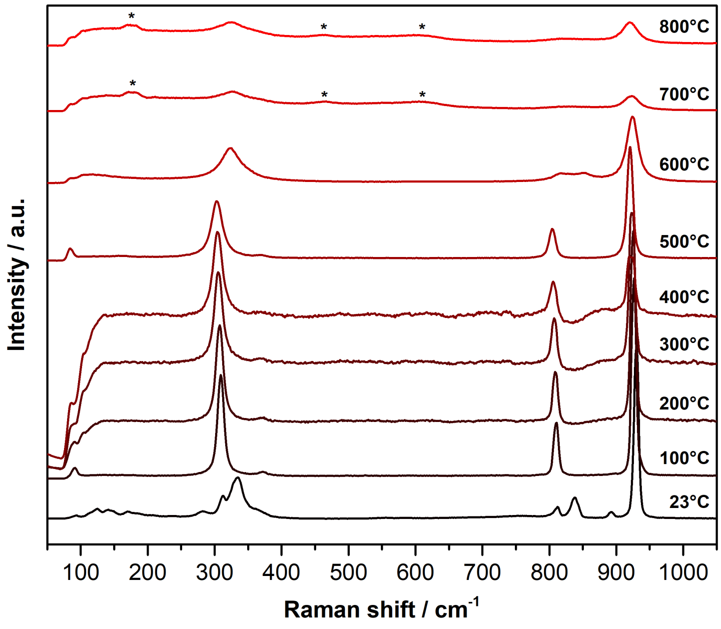

.

Figure S7. *In situ* Raman spectra of $Na_2WO_4{\cdot}2H_2O$ recorded from 23 °C to 835 °C at 457 nm (He:O_2 = 79:21, total flow = 10 mL/min, exposure time = 1.5 min, baseline-corrected); signals of the ceramic fibre carrier are indicated by an asterisk.

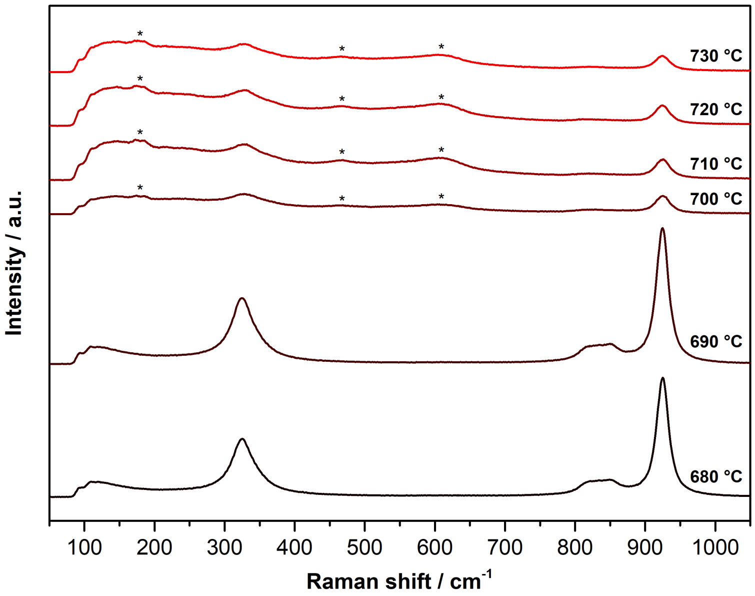

Figure S8. *In situ* Raman spectra of $Na_2WO_4{\cdot}2H_2O$ recorded from 680 °C to 730 °C at 457 nm (N_2:O_2 = 79:21, total flow = 10 mL/min, heating rate = 10 °C/min, exposure time = 5 min); signals of the ceramic fiber carrier are indicated by an asterisk.

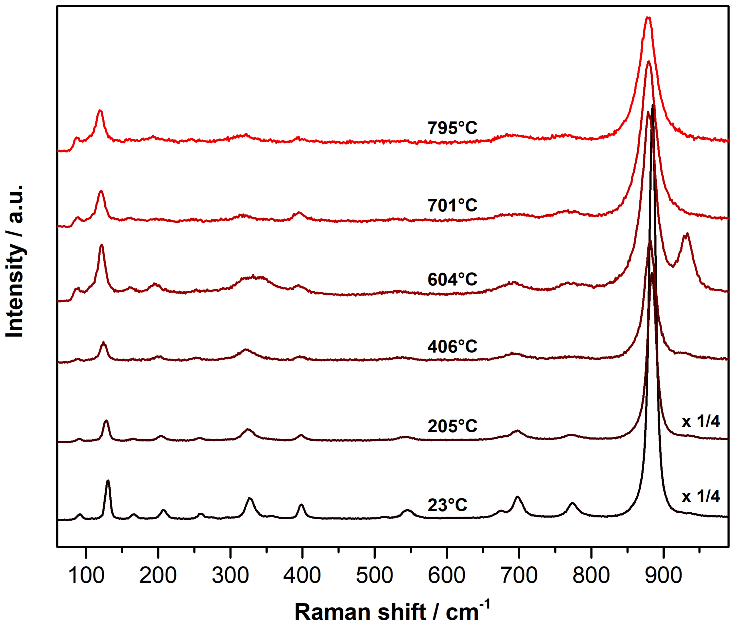

Figure S9. *In situ* Raman spectra of $MnWO_4$ recorded from 23 °C to 795 °C at 457 nm (He:O_2 = 79:21, total flow = 10 mL/min, exposure time = 0.5−3 min).

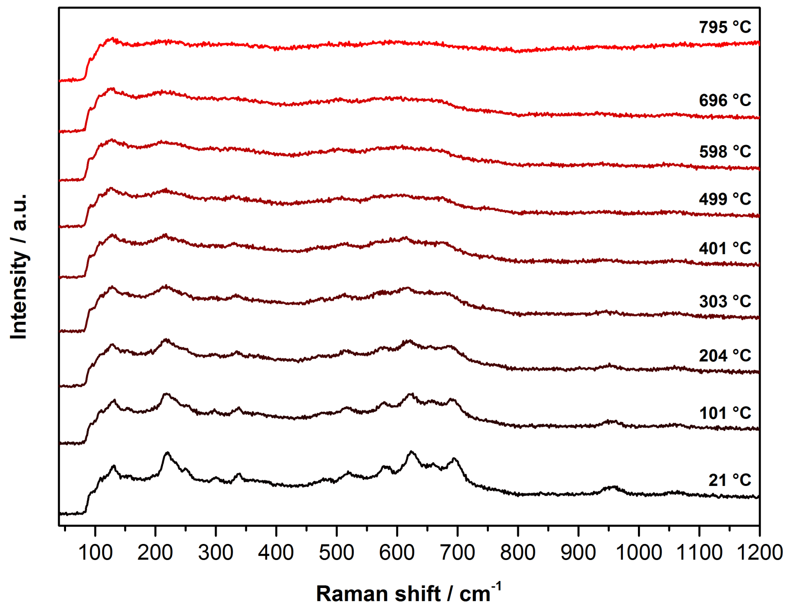

Figure S10. *In situ* Raman spectra of Mn_7SiO_{12} recorded from 23 °C to 795 °C at 457 nm (He:O_2 = 79:21, total flow = 10 mL/min, exposure time = 10 min).

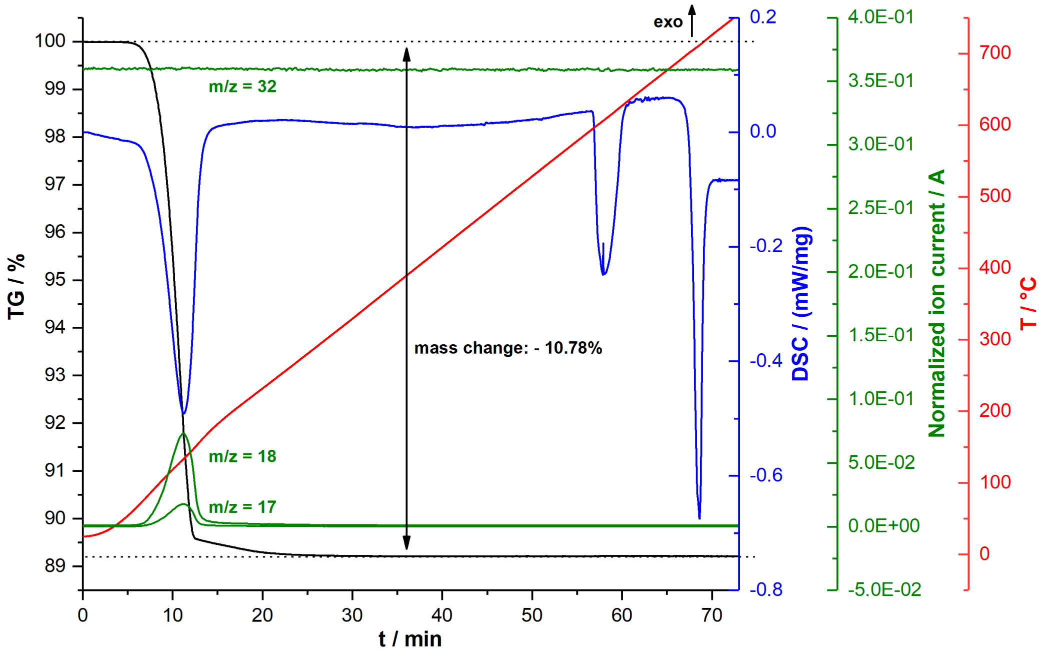

Figure S11. TG-DSC-MS of pure $Na_2WO_4 \cdot 2H_2O$ in synthetic air (21% O_2 in Ar, 100 mL/min). The sample was subjected to a heating rate of 10 °C/min to reach a maximum temperature of 780 °C. MS signal intensities were normalized to the carrier gas Ar (m/z = 40).

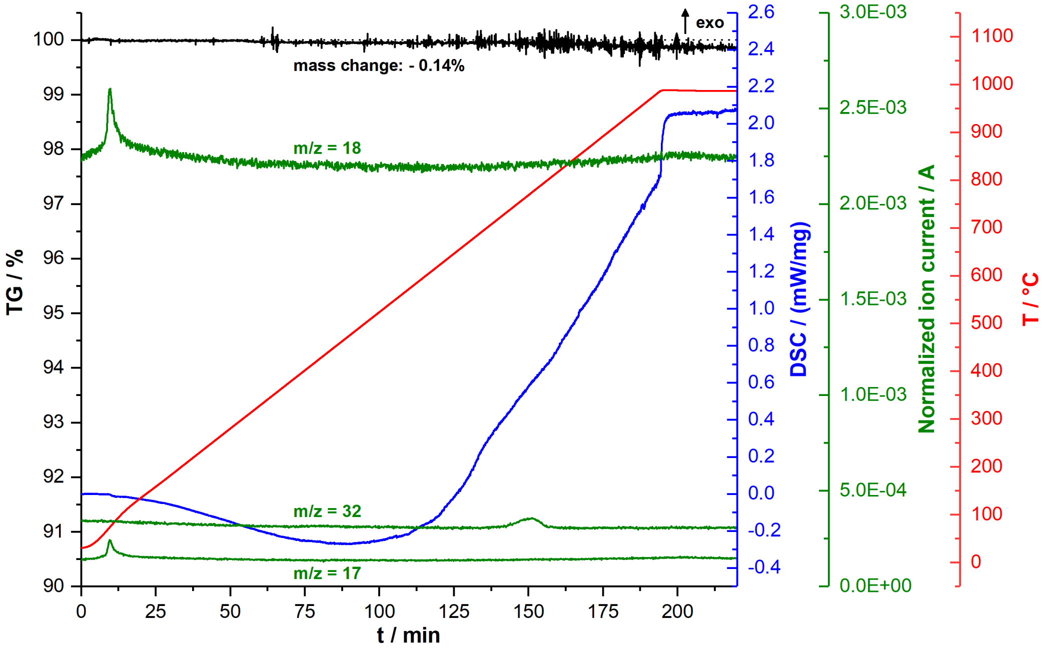

Figure S12. TG-DSC-MS of pure $MnWO_4$ in Ar (100 mL/min). The sample was subjected to a heating rate of 5 °C/min to reach a maximum temperature of 1000 °C. MS signal intensities were normalized to the carrier gas Ar (m/z = 40).

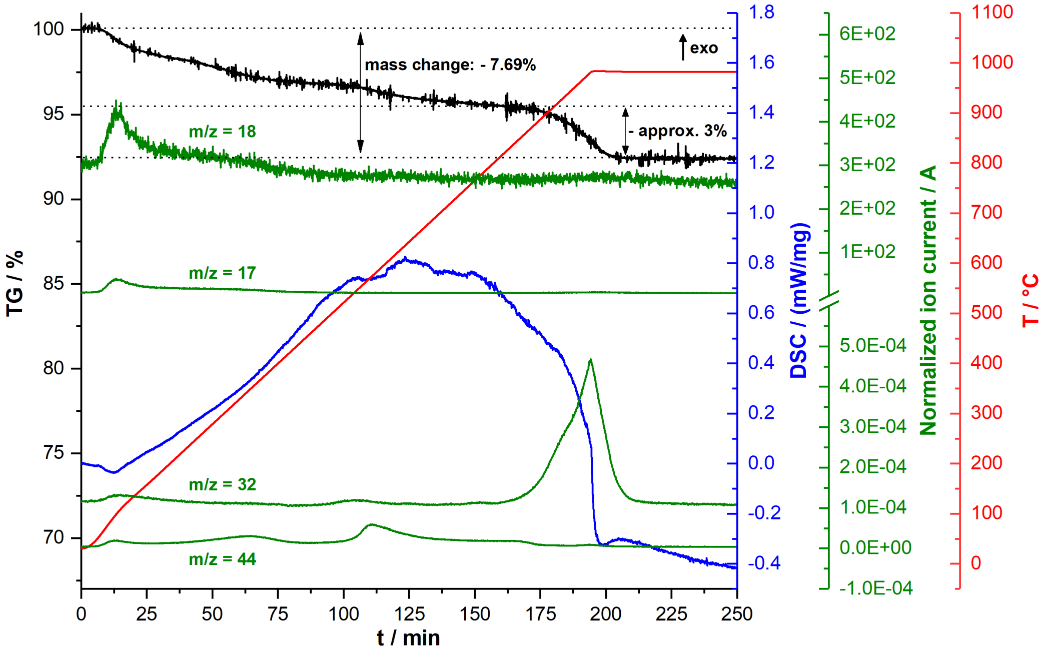

Figure S13. TG-DSC-MS of Mn_7SiO_{12} in Ar (100 mL/min). The sample was subjected to a heating rate of 5 °C/min to reach a maximum temperature of 1000 °C. MS signal intensities were normalized to the carrier gas Ar (m/z = 40).

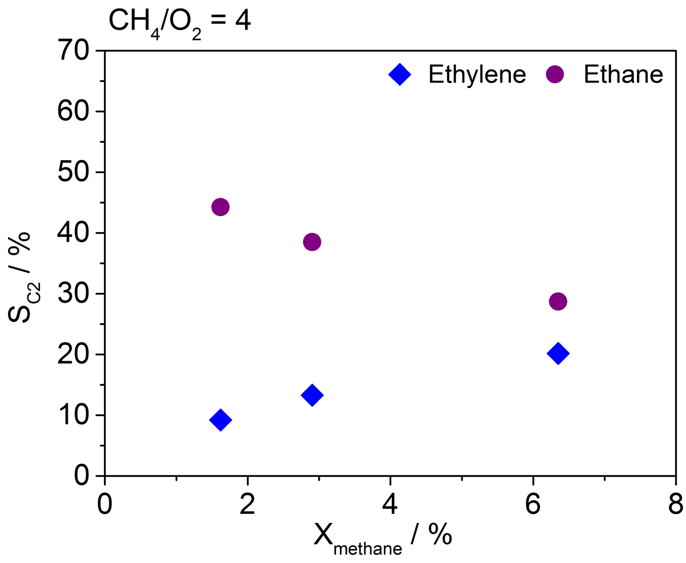

Figure S14. Performance of the Mn-Na_2WO_4/SiO_2 catalyst in the oxidative coupling of methane. A contact time variation was performed at 750 °C (W/F = 0.0025–0.0013 g*min*mL^{-1}). The measurement was performed with the undiluted catalyst to be able to compare with the operando experiment. Consequently, the yield of selective coupling products is much lower as compared to the yield measured for diluted catalysts beds.[3]

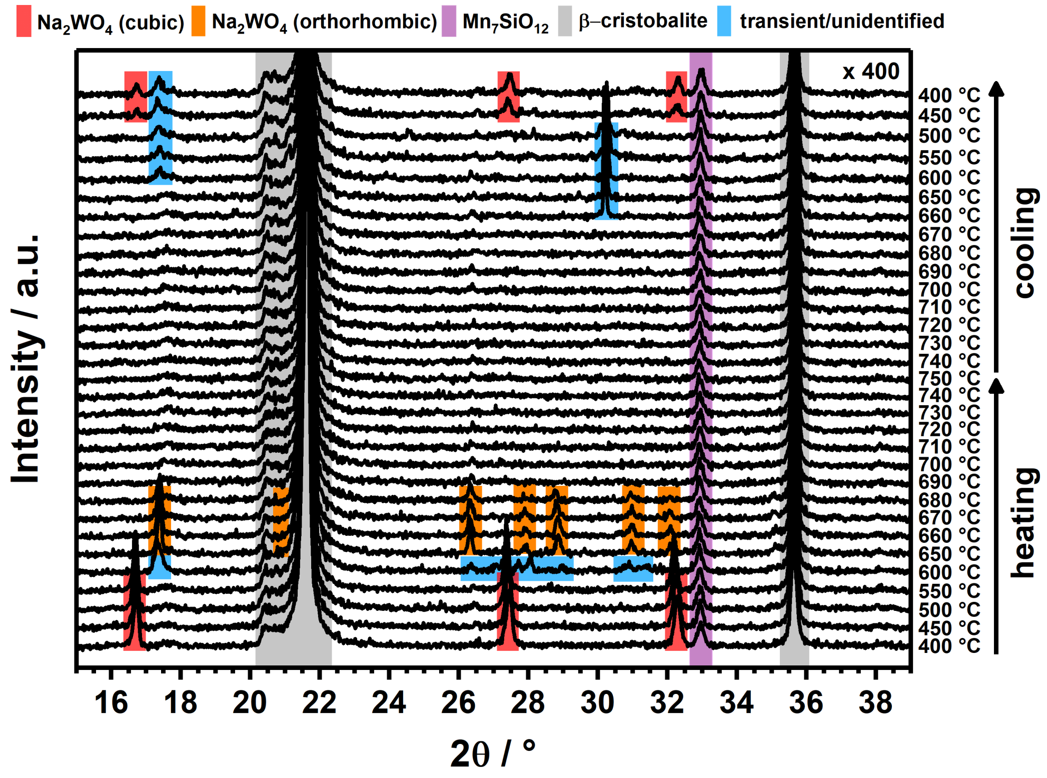

Figure S15. *In situ* XRD of the Mn-Na_2WO_4/SiO_2 catalyst under oxidizing conditions (O_2:He = 1:8, 20 mLn/min, 64.0 mg sample, W/F = 0.0032 g*min*mLn^{-1}).

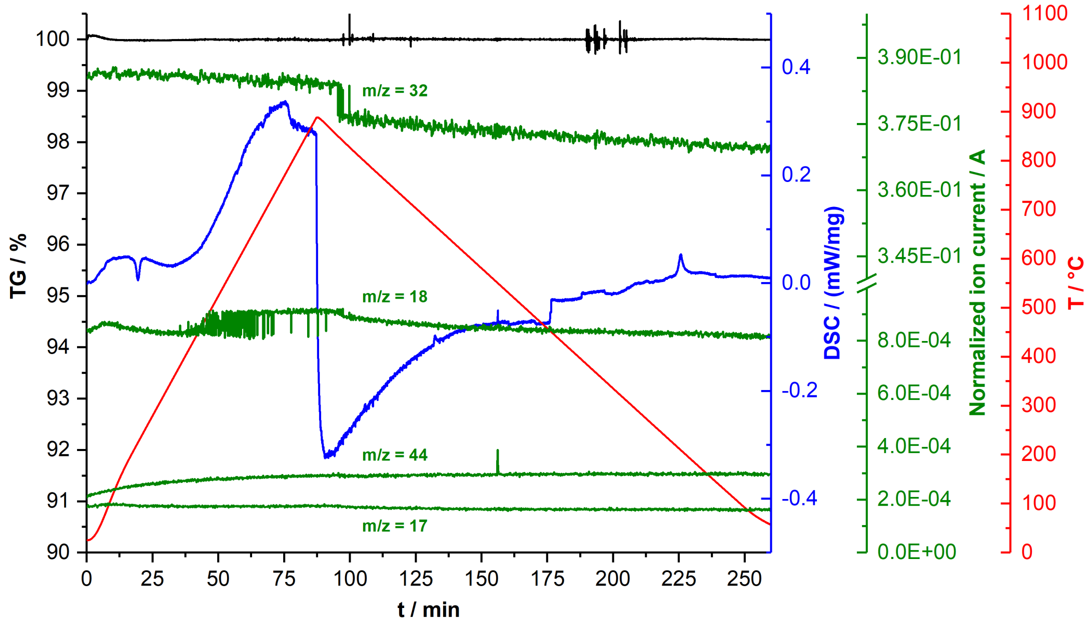

Figure S16. TG-DSC-MS of the Mn-Na_2WO_4/SiO_2 catalyst in synthetic air (Ar:O_2 = 79:21, total flow rate = 100 mL/min). The sample was subjected to a heating rate of 10 °C/min to reach a temperature of 900 °C. After reaching the maximum temperature, the sample was cooled to 30 °C at a cooling rate of 5 °C/min. MS signal intensities were normalized to the carrier gas Ar (m/z = 40).

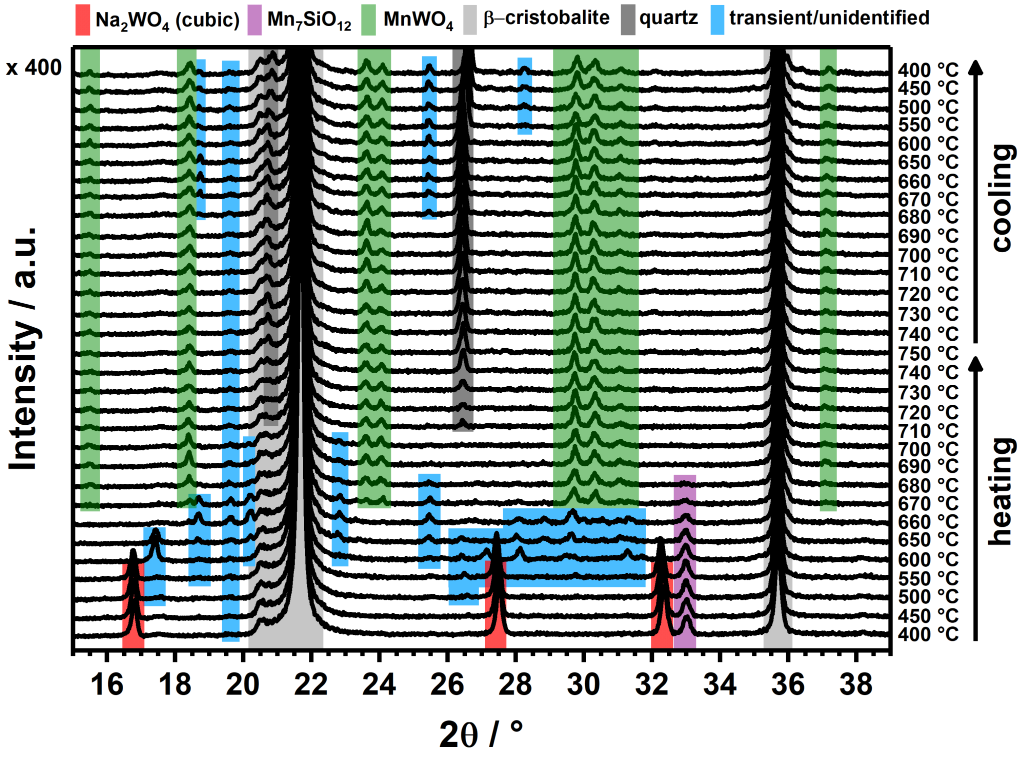

Figure S17. *In situ* XRD of the Mn-Na_2WO_4/SiO_2 catalyst under inert conditions (He, 20 mLn/min, 60.0 mg sample, W/F = 0.0030 g*min*mLn^{-1}).

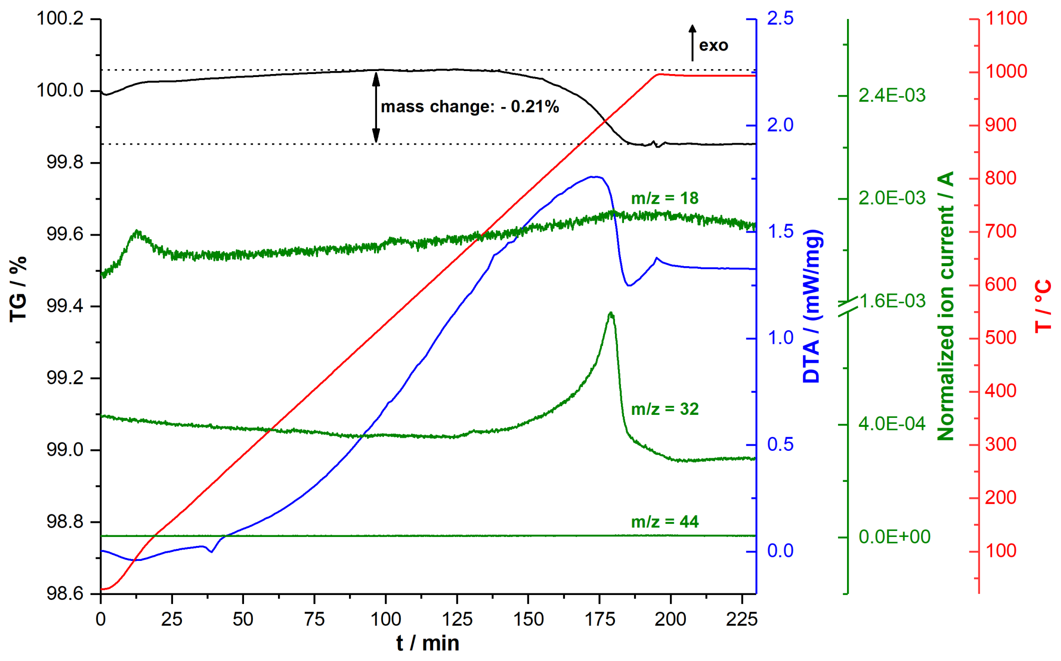

Figure S18. TG-DTA-MS of the Mn-Na_2WO_4/SiO_2 catalyst in Ar (70 mL/min). The sample was subjected to a heating rate of 5 °C/min to reach a maximum temperature of 1000 °C. MS signal intensities were normalized to the carrier gas Ar (m/z = 40).

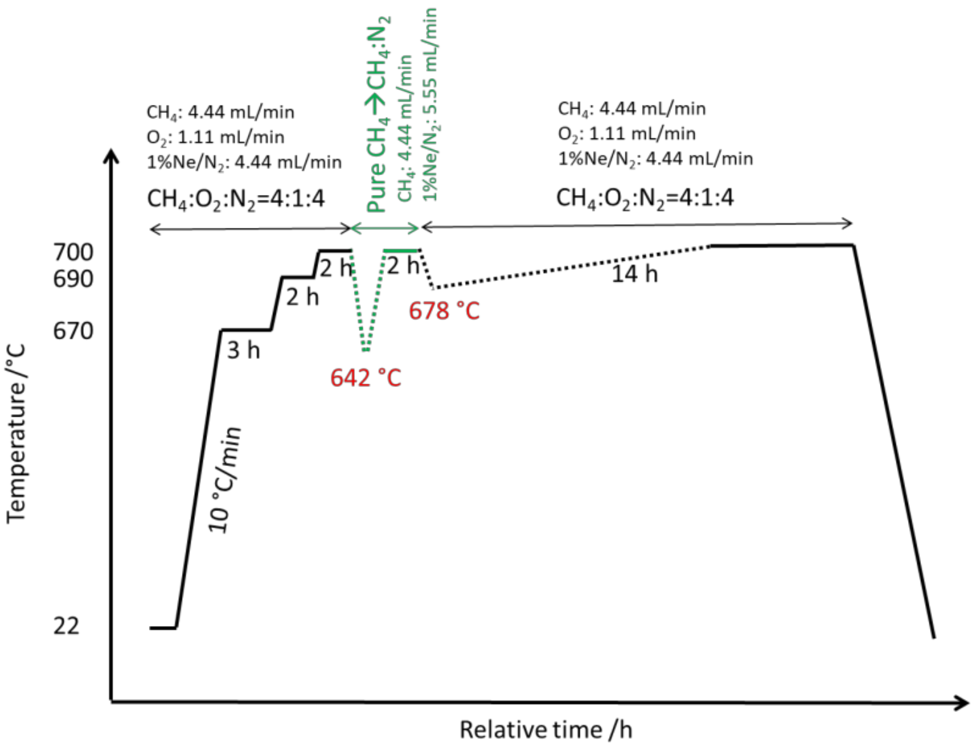

Figure S19. Temperature profile and feed compositions applied during the *operando* Raman experiment.

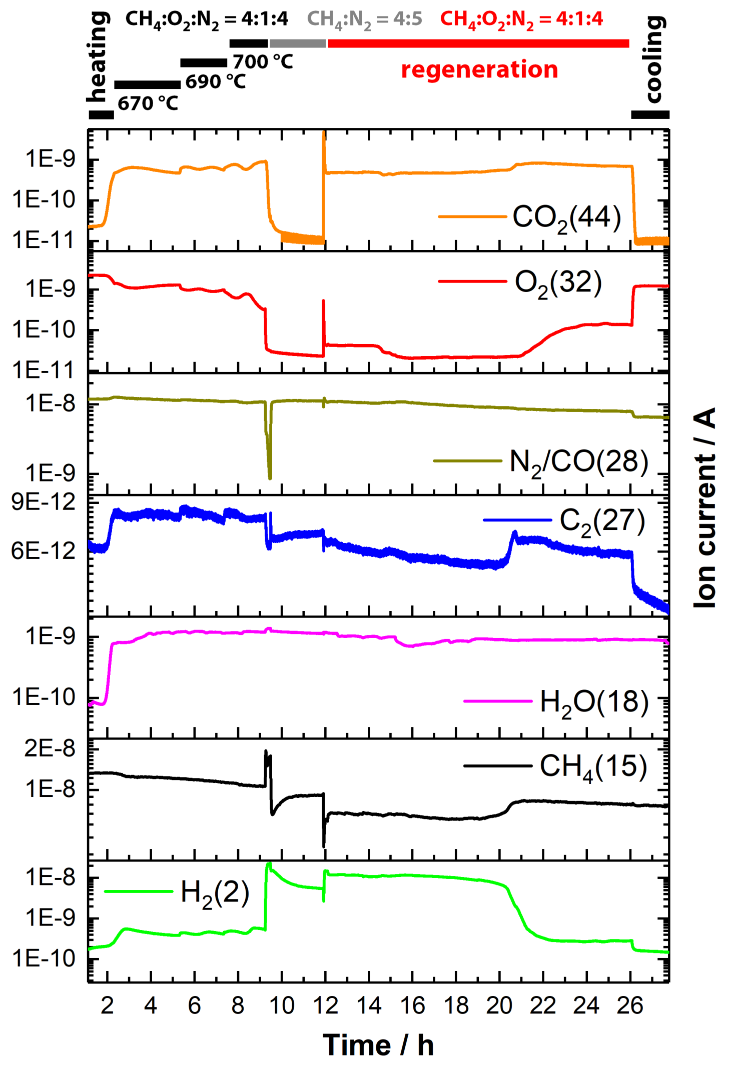

Figure S20. Mass spectrometry data recorded during the *operando* Raman experiment. The respective temperature and feed regimes are given at the top of the figure.

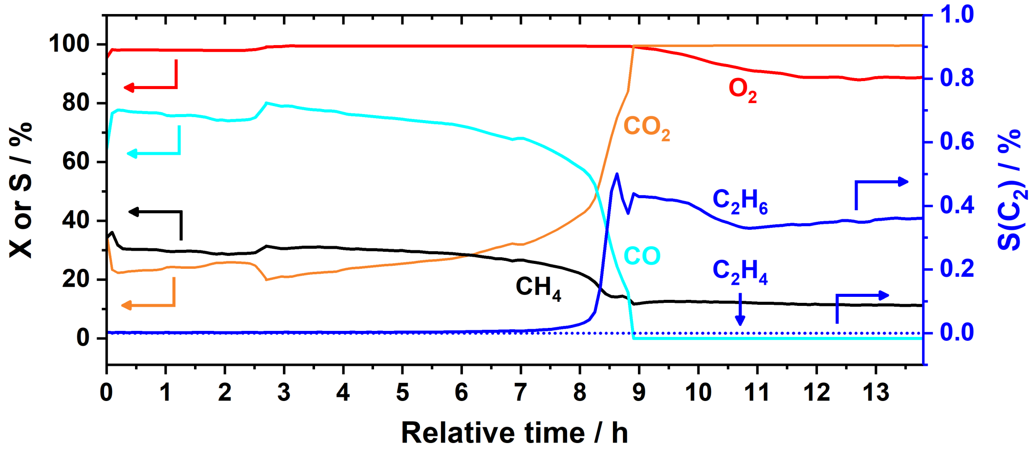

Figure S21. Gas chromatography data recorded during catalyst regeneration in $CH_4/O_2/N_2$ = 4:1:4 at 700 °C.

6 References

[1] R. Hochleitner, natural mineral braunite, Langban, Sweden ed., Mineralogische Staatssammlung München.

[2] a) S.-f. Ji, T.-c. Xiao, S.-b. Li, C.-z. Xu, R.-l. Hou, K. S. Coleman, M. L. H. Green, *Applied Catalysis A: General* **2002**, *225*, 271-284; b) S. Ji, T. Xiao, S. Li, L. Chou, B. Zhang, C. Xu, R. Hou, A. P. E. York, M. L. H. Green, *Journal of Catalysis* **2003**, *220*, 47-56.

[3] S. A. Akhade, J. R. Kitchin, *The Journal of Chemical Physics* **2012**, *137*, 084703.
